# Effects of classrooms’ architecture on academic performance in view of telic versus paratelic motivation: a review

**DOI:** 10.3389/fpsyg.2015.00746

**Published:** 2015-06-03

**Authors:** Peter Lewinski

**Affiliations:** Amsterdam School of Communication Research, University of Amsterdam, Amsterdam, Netherlands

**Keywords:** academic performance, learning outcomes, learning environment, classroom design, reversal theory

## Abstract

This mini literature review analyzes research papers from many countries that directly or indirectly test how classrooms’ architecture influences academic performance. These papers evaluate and explain specific characteristics of classrooms, with an emphasis on how they affect learning processes and learning outcomes. Factors such as acoustics, light, color, temperature, and seat arrangement are scrutinized to determine whether and by how much they improve or hinder students’ academic performance in classrooms. Apter’s (1982, 1984, 2014) reversal theory of telic versus paratelic motivation is presented and used to explain these findings. The results show preference for a learning environment that cues a telic motivation state in the students. Therefore, classroom features should not be distracting or arousing. Moreover, it appears the most influential factors affecting the learning process are noise, temperature and seat arrangement. In addition, there is no current agreement on how some particular physical characteristics of classrooms affect learning outcomes. More research is needed to establish stronger conclusions and recommendations.

## Telic and Paratelic Motivation

A substantial body of theories already exists on how people evaluate their environments, what elements they prefer, how they interact with physical surroundings and many other related aspects. Especially relevant to a classroom environment would be the approach developed by Apter (1982, 1984, 2014), who describes two types of motivation—telic and paratelic within reversal mode-based theory of motivation (and personality). Telic motivation is oriented toward achieving a goal, and people in this state look for low arousal environments with an end state being relaxation. On the other hand, paratelic motivation is focused on the activity itself and is triggered by lack of arousal (boredom), therefore heightened arousal is pleasant and people are motivated to seek highly arousing environments when in this motivational state. In telic mode, means-ends motivations are reactive, goal-oriented and end-oriented, whereas in paratelic mode, the motivations are proactive, behavior-oriented and process-oriented. Thus, the assumption of this theory is that students—or anyone attempting to acquire knowledge—would much rather prefer environments that facilitate experiencing a telic state. On the contrary, environments evoking paratelic states would be undesirable as classrooms, as these would not motivate occupants toward essential goals. It is understood that a myriad of theories and frameworks are available for creating well-designed learning environments, e.g., the *Gestalt* laws of perceptual organization (Wagemans et al., 2012) or *Ergonomic Classroom Assessment* (Harik and Fattouh, 2010). However, here the focus will be how telic versus paratelic motivation may explain the results of particular research findings.

## Method

In following passages, we seek to answer the question of which physical factors of a typical classroom affect academic performance, and in what way. We hope to determine whether it is possible to establish which elements play the most important role, which ones play smaller roles and if there exists disagreement among environmental psychologists regarding this topic. In the following order, these classroom characteristics will be evaluated: (a) acoustics; (b) light; (c) color; (d) temperature; and (e) seat arrangement. In the end, we will show how each one affects the learning process and academic achievement in light of the telic motivation framework.

## Acoustics

Noise is well known to have an impact on human performance. Chiang and Lai (2008) investigated and identified some of the negative effects of working in a noisy room, with a focus on young children. They claim that noise influences not only learning outcomes, but also the health of the occupants. In the case of young children, they have not yet developed enough executive skill in activities involving communication channels, like speech comprehension, use of language, and written and oral skills (Mills, 1975). Therefore, interference profoundly interrupts the process of acquiring those essential capacities in children, and noise is far from the only possible kind of interference. Noise undermines reading, writing and comprehension skills, as well as overall academic performance, as noise makes it hard to focus on the task being performed (DiSarno et al., 2002). Chiang and Lai (2008) reviewed previous findings on noise’s harmful effect on mental and physical wellbeing as part of their study. From a plethora of demonstrable effects, the following negative outcomes were reported specifically in the context of a noisy room: getting tired easily, leading to lower efficiency; increased heart rate; dyspepsia; poor appetite; insomnia; headache; tinnitus; and facial pallor (p. 1621).

Zannin and Zwirtes (2009) carried out a study comparing schools built in 1977–2005 according to three different recommended standard designs for school buildings. Reverberation time, sound insulation coefficients and ambient noise were correlated to international standards. Their research confirms what previous studies have found. Many classrooms are simply not comfortable places to acquire knowledge or to be mentally focused at all time, due to noise interference. Zannin and Zwirtes (2009) show that even following standard best practices for design, the results are sub-optimal for a learning environment. Most importantly, the authors highlight that the relative position of schoolyards and recreation spaces is often ill conceived with respect of the rest of the school. In addition, the architectural design and material choices allow for voice and noise to be carried between two adjoined classrooms and hallways.

Noise level is another important issue when looking at how acoustics affects academic performance. No internationally recognized norms on maximum noise levels for classrooms exist, but, for example, Brazil’s regulatory body has mandated a maximum of 40 dB(A) (Zannin and Marcon, 2007). However, one well-controlled study of classroom noise levels revealed values over 40 dB(A) for each of five tested classrooms with open and closed windows (Zannin and Marcon, 2007). In the same study, the authors found that both students and teachers pointed out that noise in the classroom was a major source of disturbance for them. Interviews with 62 teachers and 462 students included questions pertaining to how they evaluated various acoustic aspects of their classrooms. These interviews indicated that bothersome noise came mostly from other classrooms. Presumably, teachers and students in adjoining classrooms spoke too loudly. The study reported that every objectively measured acoustic characteristic of the classrooms (background noise, reverberation time, sound insulation) fell short of Brazil’s standards. In yet another study, researchers showed clearly that classrooms were not a productive and comfortable place to acquire knowledge, because of poor acoustics (Kruger and Zannin, 2004). Zannin et al. (2012) and Zannin et al. (2013) recently found this pattern of negative effects again.

## Light

The quality and quantity of light (illumination) undoubtedly influences the perception of comfort in a particular space. Illumination has strong and well-documented effects, but less obvious is the case of light quality. Boray et al. (1989) undertook a study evaluating how different types of lighting (warm white, cool white, and full-spectrum fluorescent) affect various dependent variables, including: cognitive performance, room attractiveness, judged room size, and pleasure of room. They found no significant differences among all dependent variables with respect to the type of lighting used. The researchers could only conclude that management prefers warm white or cool white over full-spectrum light, chiefly because the first two are less costly to buy and maintain.

A natural assumption might be that more light always creates a better, more positive impression of a classroom’s qualities. However, one study clearly shows an upper limit to classroom lighting, above which the lighting has negative effects. Kruger and Zannin (2004) conducted a study in Brazil comparing luminance in classrooms throughout the course of several days in August 2000. One room was equipped with windows with light shelves; another was not. Classrooms were on the same side of the building, and all other variables were held constant. Interestingly, these studies showed that rooms both the with light shelves and without light shelves condition had advantages and disadvantages. In late afternoon, windows with light shelves produced light below prescribed luminance, whereas windows without light shelves created high luminance values throughout the day, which can lead to gradual furniture and fixture damage—and distract students and teachers—as well as increase thermal discomfort. This research shows that even such feature like light shelves might have some drawbacks.

## Color

The effects of exposing people to particular colors have always intrigued scientists. Color most certainly affects our experience of the world. For instance, an ongoing debate concerns the peculiarly named color “baker-miller pink,” which is purported to lower stress and anxiety levels, as well as affecting physiological functions—e.g., reducing blood pressure and pulse rate (Schauss, 1985; Profusek and Rainey, 1987; Bennett et al., 1991). As far back as 1988, Gilliam and Unruh noted that the results of studies on baker-miller pink were incongruent with each other. Therefore, Gilliam and Unruh (1988) investigated the topic themselves, finding no significant differences between peoples’ experience of and reactions to ordinary white walls and the more unusual baker-miller pink walls.

Elliot et al. (2007) exposed participants to the color red, green, or black before giving them a test; they found that exposure to red, even if participants were not consciously aware of the exposure, impaired their academic performance. The effect was found even when a number was written in red ink at the top of a sheet of paper. Greater right frontal hemisphere EEG activation was found when students were exposed to red, which is consistent with similar findings of greater activation in right frontal relative to the left frontal cortex following exposure to the color red.

Another argument for the negative effects of the color red pertains to findings by Gimbel (1997) and Pile (1997), which are summarized in a table as part of their research paper (Gimbel, 1997; Pile, 1997 as cited in Fisher, 2005). Notably, these authors suggest that the color green is best for classrooms. Gimbel (1997) and Pile’s (1997) table also suggests which colors might be responsible for specific student behaviors. For example: red—alert, increased pulse, activity; green—balance, judgment, arrested movement, stasis. However, in his book on environmental psychology, Gifford (2007) argues that performance on math and reading tests did not vary among students who performed in classrooms with different colored walls.

In a brief review of how to design effective study environments, Stone (2001) highlights the lack of a clear relationship between color and mood (working from the assumption that mood is directly connected to performance). Based on a review of dozens of studies, Stone observes that if any relationship does exist, the most likely associations are red and yellow colors with stimulation and blue and green colors with calming effects. Stone also found out that color did have an impact on qualitatively different tasks (math task versus reading task). The color of the surrounding environment affected performance on more difficult tasks, i.e., the reading task. A further finding was that the lowest performance on cognitively demanding tasks was in classrooms with red walls.

## Temperature

We argue that temperature plays a significant role in how likely we are to feel comfortable while performing a task. Probably the ideal temperature is one that is hardly noticeable—neither too cold nor too hot. Unsurprisingly, the temperature of classrooms is another important factor that contributes to students’ academic performance. In a literature review of thermal quality and students’ learning, Earthman (2002) highlighted the existence of prime temperature ranges for optimal learning outcomes. Generally, research shows that temperatures between 68 and 74°F—20 and 24°C—are most conducive to comfort and, by extension, learning. In addition, 50% relative humidity was found to be an acceptable value for classrooms (Earthman, 2002). A link between temperature and acoustics exists, ill-maintained air conditioning systems, beside obvious problems with maintaining optimal classroom temperature, may produce considerably uncomfortable noise.

## Seat Arrangement

We argue that the seat arrangement is a potent means to efficiently manipulate the physical characteristics of the classroom to ensure high performance of both students and teachers. Douglas and Gifford’s (2001) research incorporated a lens model approach (“a probabilistic representation of the way perceivers use environmental cues to draw inferences about the environment,” p. 296), which was originally developed by Brunswik (1956). Students and professors, who evaluate classroom physical characteristics, might not at first glance be related to issues of academic performance. However, Douglas and Gifford’s (2001), at the outset of their study modified a lens model to suit their needs. Students and professors in this study judged how friendly the classroom was and how much they preferred it. Douglas and Gifford (2001) explain how friendliness and overall preference was described on the questionnaire. Friendliness was defined as “(…) how warm, comfortable, etc., the room makes you feel, in your own opinion.” Overall preference was defined as “a global rating of all factors that you consider important to the classroom environment” (p. 298). Each participant was shown two photos of 35 various classrooms, and she evaluated them on the scale just described. Surprisingly, only three characteristics of the classroom explained between 40 and 57% of the variance in the evaluation of friendliness and overall preference by both students and professors. In this study, both groups preferred sociopetal arrangements of seats. Sociopetal arrangement is defined as a placement of chairs and tables in a way that it allows for a greater social interaction amongst students and professors. Two other notable properties were a view of the outdoors and comfortable seats. Not surprisingly, quality of seating was more significant for students, as teachers tend to have comfortable seats owing to their higher status. Douglas and Gifford (2001) pointed out that users of classrooms did not rate highly such classroom properties as brightness, room size and aesthetic complexity.

Douglas and Gifford’s (2001) investigation offers no insights regarding how these various classroom properties are related, nor if they individually or together actually relate to the learning process. However, we argue that it is reasonable to assume that physical characteristics known to elicit positive feelings and make people comfortable in the learning environment must necessarily be correlated with stronger student performance. Being in an appealing classroom, therefore, is far preferable to being in a classroom without sociopetal seating arrangements, a view to the outdoors, and comfortable seats. This assertion remains to be tested, however.

Rosenfield et al. (1985) tested how desk and chair arrangement affected students’ behavior. Elementary school children were measured according to their on-task behaviors, such as hand-raising, discussion comment, questioning/pupil request, listening, out-of-order comment, and speaking; and on their off-task behaviors, such as disruptive conduct, withdrawal, and aggression. The dependent variables mentioned above were clearly defined and measured by trained evaluators. The possible desk arrangements were clusters, rows, and circles. Results showed that students seated in circles showed the most on-task behaviors. The second-best arrangement of desks and chairs was a cluster arrangement, and the least effective was desks arranged in rows. As expected, such variables as sex, age, and attitude toward studying affected students’ scoring, too.

## Conclusion

We hope to have offered up an insightful review of the literature and suggested new avenues for study. Inasmuch as the perception of learning environments is invested with desires to complete planned actions, students ideally should experience a telic motivation state (seek low arousal, because they are goal oriented). Pleasant, relatively unstimulating and non-arousing environments must therefore be provided. The finding that the color red is arousing and leads to decreased performance in academic settings, along with many other factors outlined below that have drastic performance impacts, is congruent with the predictions of Apter’s (1984, 2014) telic motivation within reversal theory.

Perhaps not surprisingly, factors that most significantly affect the learning process are noise, temperature, and—somewhat unexpectedly—seating arrangement. In many cases, noise level exceeded international standards, profoundly disturbing staff members and students, not to mention that distracting noise levels have been found to impair childhood development and directly influence one’s health. Temperature and humidity have less dramatic consequences in the learning environment, but if temperature is not maintained at a comfortable level (between 68 and 74°F), this variable may negatively affect students’ performance. The literature on this topic once more supports the classical view of “room temperature” to be valid and supported empirically. One important factor contributing to better temperature control is a well-maintained air conditioning system; however, that same system can increase ambient noise to uncomfortable levels, demonstrating the interactions between different variables. A good compromise between comfortable temperature level and noise level must be discovered, because in general, air conditioning system noise is taken for granted in order to keep in classrooms at a proper temperature.

In the light of Apter’s reversal theory of telic versus paratelic motivation, an easy argument could be made that noise creates a distracting environment, which is not conducive to a telic state in students who wish to focus on a task. Especially prone to decreases in performance are younger children, who usually have not yet developed good coping mechanisms with interference like noise. These students cannot stay long in telic mode if such disturbances are present and persistent in the surrounding environment. In addition, temperature affects the ability to stay focused and goal-oriented, as excess cold or warmth diverts one’s attention toward how to resolve the uncomfortable state instead of focusing on a task. A seemingly incoherent finding regarding telic versus paratelic motivation states is that people appear to prefer sociopetal seat arrangement, which fosters paratelic rather than telic motivation. Seat arrangement that encourages social interaction feeds directly into paratelic goals, such as talking to fellow students and generally being aware of others, which unavoidably diffuses one’s attention. On the other hand, however, human beings are such highly social animals that isolation may actually provoke a paratelic state as part of attempting to decrease the uneasiness felt when opportunities to interact with people are limited. The likely explanation here is that it is preferable to be distracted by others rather than having no opportunity to interact with them, therefore experiencing a lower activation threshold for the paratelic motivation state.

In regards to seat arrangement, a design that provides many opportunities for social interaction is preferred by both students and teachers. However, numerous individual factors play a role, too. Tanahashi (2007) notes that flexibility in seating is important in that it allows for adapting to shifts in teaching styles. In addition, Martin (2002) mentions that the majority of teacher-centered teachers state there is little relationship between their pupils’ learning outcomes and the physical environment where learning happens. However, child-centered teachers claim just the opposite. Insofar as results suggest the benefits of arranging seats in sociopetal configuration and using comfortable seats for both students and teachers, it should equally be mentioned that teaching styles adopted at particular moments must also be considered. However, one might argue that students should not be too comfortable, as excess relaxation could result in a lack of focus. One idea worth investigating is adjustable, ergonomic chairs; while potentially an interesting possibility, the higher cost of such chairs could be a deal breaker for many schools, even if they did prove to positively influence learning outcomes. Last, we provide a suggestion for future research that certain types of learning (e.g., involving creativity or body movement) will benefit most from arrangements that cue paratelic state.

### Conflict of Interest Statement

The author declares that the research was conducted in the absence of any commercial or financial relationships that could be construed as a potential conflict of interest.
